# A defined medium to investigate sliding motility in a *Bacillus subtilis *flagella-less mutant

**DOI:** 10.1186/1471-2180-6-31

**Published:** 2006-03-17

**Authors:** Ray Fall, Daniel B Kearns, Tam Nguyen

**Affiliations:** 1 Department of Chemistry and Biochemistry, University of Colorado, Boulder, CO 80309-0215, USA; 2 Department of Molecular and Cellular Biology, Harvard University, 16 Divinity Ave., Cambridge, MA 02138, USA; 3 Department of Biology, Indiana University, Bloomington, IN 47405-7000, USA

## Abstract

**Background:**

We have recently shown that undomesticated strains of *Bacillus subtilis *can extensively colonize the surfaces of rich, semi-solid media, by a flagellum-independent mechanism and suggested that sliding motility is responsible for surface migration. Here we have used a flagella-less *hag *null mutant to examine and confirm sliding motility.

**Results:**

Using a defined semi-solid medium we determined that a *B. subtilis hag *mutant colonized the surface in two stages, first as tendril-like clusters of cells followed by a profuse pellicle-like film. We determined the levels of macro- and micro-nutrients required for the tendril-to-film transition. Sufficient levels of each of the macronutrients, glycerol, Na-glutamate, and Na-phosphate, and inorganic nutrients, K^+^, Mg^2+^, Fe^2+ ^and Mn^2+^, were required for robust film formation. The K^+ ^requirement was quantified in more detail, and the thresholds for complete tendril coverage (50 μM KCl) or film coverage (2–3 mM KCl) were determined. In addition, disruption of the genes for the higher affinity K^+ ^transporter (KtrAB), but not the lower affinity K^+ ^transporter (KtrCD), strongly inhibited the formation of both tendrils and films, and could be partially overcome by high levels of KCl. Examination of *hag *tendrils by confocal scanning laser microscopy revealed that tendrils are multicellular structures, but that the cells are not as highly organized as cells in wild-type *B. subtilis *pellicles.

**Conclusion:**

These results suggest that *B. subtilis *can use sliding motility to colonize surfaces, using a tendril-like growth mode when various macronutrients or micronutrients are limiting. If nutrients are balanced and sufficient, the surfaces between tendrils can be colonized by robust surface films. Sliding motility may represent a strategy for nutrient-deprived cells to colonize surfaces in natural environments, such as plant roots, and the media described here may be useful in investigations of this growth phenotype.

## Background

Bacteria use a variety of motility mechanisms to colonize environments, including flagella-dependent swimming and swarming, and flagella-independent, twitching, gliding, and sliding (reviewed in [1]). Of these motility mechanisms, the least investigated is sliding motility, which Henrichsen [2] defined as surface translocation produced by expansive forces in the growing colony combined with special surface properties to lower the friction between the cells and substrate. Harshey [1] points out that sliding motility is a passive mode of translocation for spreading over surfaces. Sliding motility has been genetically studied in *Mycobacterium smegmatis *and found to require the formation of acetylated glycopeptidolipids (GPLs) in the outermost layer of the cell envelope [3,4]. It has been proposed that the hydrophobic fatty acyl tails of GPLs on the cell surface lower the friction to produce sliding motility on the surface of the medium (i.e. agarose). Additional examples of sliding-type motility have been reported in other bacteria, but not investigated in detail (reviewed in [1]).

Wild strains of *Bacillus subtilis *are known to translocate over solid surfaces by a mechanism of swarming motility [5,6]. Swarming cells secrete a lipopeptide surfactant, called surfactin, to reduce surface tension and motility is powered by rotating flagella [5,7,8]. We have recently reported that wild type *B. subtilis *strains can rapidly colonize the surface of semi-solid media in a flagellum-independent manner, and suggested that sliding motility might play a role [9]. It was shown that such surface colonization was also dependent on the secretion of surfactin, but microscopic examination of the edges and interior cells of sliding surface colonies did not reveal abundant flagella. We suggest that *B. subtilis *has two distinct modes of surface translocation, swarming and sliding, which are presumably advantageous under different environmental conditions.

Here we have developed an experimental system in *B. subtilis *to study sliding motility. Two key components of this approach are the use of i) a flagella-less mutant to rule out the contribution of swarming in surface migration, and ii) a defined growth medium that was earlier developed to study the formation of floating pellicles and fruiting bodies in *B. subtilis *[10]. The defined medium allowed us to manipulate the essential macro- and micronutrients needed for sliding motility and colony spreading. As with some gram-negative bacteria, such as *Pseudomonas aeruginosa*, which can swim, swarm or show twitching motility [11], the work reported here suggests that *B. subtilis *also has multiple means of colonizing surfaces.

## Results

### A defined medium to visualize K^+^-dependent sliding motility in a hag mutant

As mentioned above, we have presented evidence that undomesticated *B. subtilis *strains, such as the Marburg strain 3610, can colonize the surfaces of semi-soft media using flagellar-dependent swimming and swarming as well as flagellar-independent sliding motility. To focus on sliding motility, a *hag *null mutant (defective in the coding gene for flagellin, an essential subunit in flagellum assembly [12]) of the undomesticated 3610 strain was used to eliminate any contribution of flagellar-dependent motility. Furthermore, a defined medium (MSgg) that has been used to study pellicle and fruiting body formation in *B. subtilis *[10] was modified to control the level of potassium ion (K^+^), as this monovalent cation is essential for flagellum-independent surface colonization by undomesticated *B. subtilis *[9]. For the modified medium, termed MSggN, the potassium phosphate component was substituted by equimolar sodium phosphate, and potassium ion levels were determined by the amount of KCl added.

**Figure 1:**
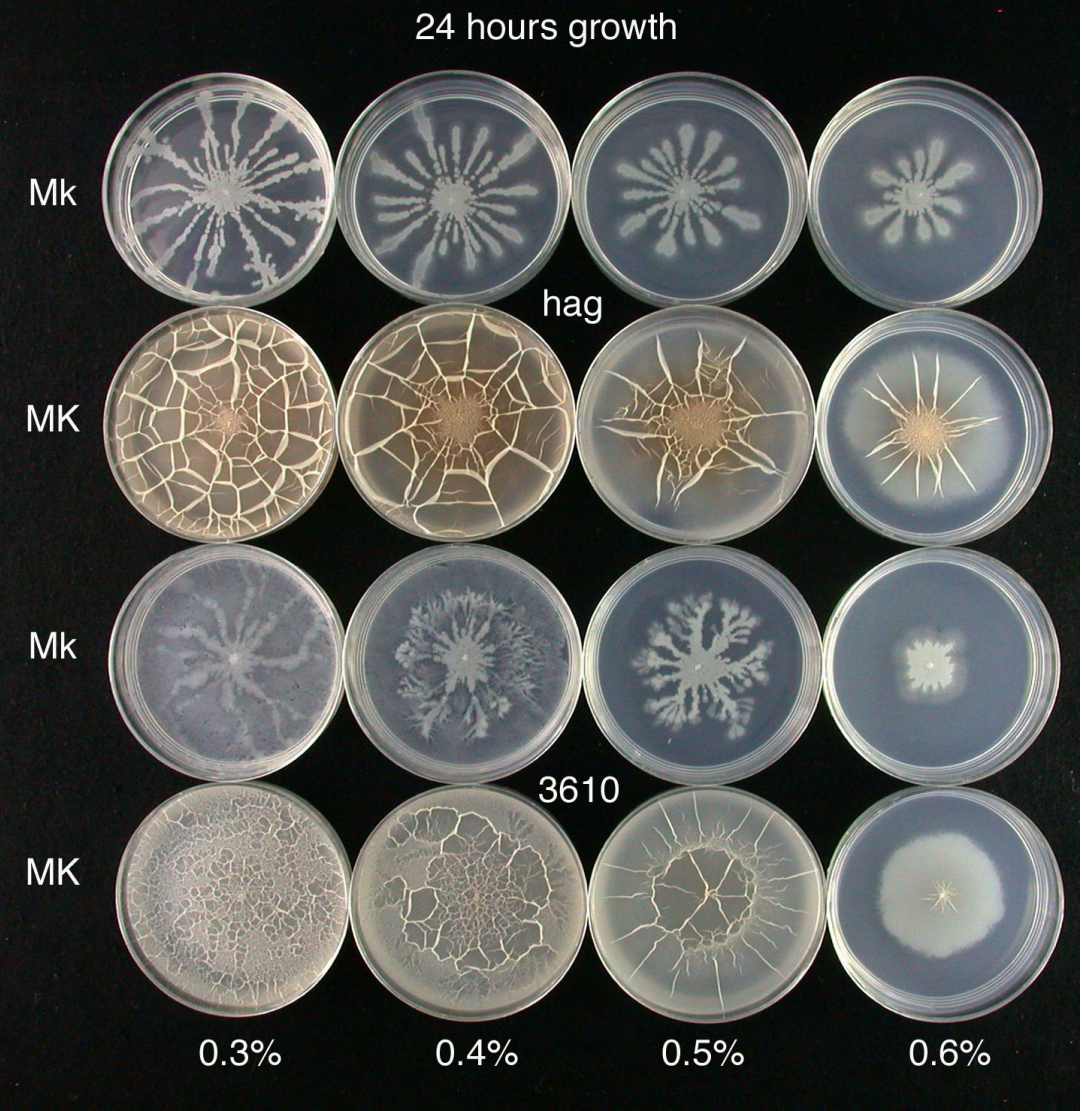
**Surface colonization by a *B. subtilis hag *mutant DS64 and its wild type parent (3610) on defined semi-solid media is dependent on the level of potassium ion and agarose concentration**. Semi-solid MSggN agarose plates (0.3 to 0.6% w/v agarose) were inoculated in the center with sharpened toothpicks with both *hag *and 3610 strains (in triplicate). After growth for 18 h at 37°C, typical plates were photographed. Abbreviations: Mk, MSggN medium with 100 μM KCl; MK, MSggN medium with 5 mM KCl.

As shown in Fig. 1, growth of the *hag *mutant on MSggN agarose plates from the central point of inoculation occurred by means of long tendril-like arms if the KCl level was low (i.e. 100 μM). In contrast, if plates contained 5 mM KCl the surface was completely colonized after 18 h of growth (37°C); and the surface had a wrinkled, web-like appearance that is similar to that seen in *B. subtilis *pellicles that form on the surface of MSgg liquid medium [10,13]. In contrast, the parent 3610 strain swarmed over the surface with a more dendritic central colony (low KCl) and produced less robust surface films (high KCl). The rate of surface migration of the swarming 3610 cells was about twice that of the sliding *hag *cells. Notably, the sliding motility seen in the *hag *mutant was dependent on the secretion of surfactin, as a *hag *mutant containing a surfactin gene deletion (strain RFH1) did not spread significantly from the point of inoculation, but the colony could spread if authenthic surfactin (50–200 μg) was first added to the center of the plate.

Other investigations of bacterial surface motility [1] have demonstrated that the degree of colony spreading is often highly dependent on the concentration of the solidifying agent, and can be dependent on the method of inoculation. We examined these issues, and, as shown in Fig. 1, as the concentration of agarose was increased the degree of surface colonization – either in *hag *or 3610 cells – decreased. To test the effect of the method of inoculation on surface growth of the *hag *mutant, agarose plates (in triplicate) were inoculated either by toothpick from LB (MLS) agar plates or by centrally spotting with 2.5 μl of various cell densities from cells grown in LB (MLS) broth. Qualitatively, the appearance of tendrils or films on each of these plates was independent of inoculation method or cell density, if at least 5 × 10^3 ^viable cells were spotted (data not shown), and the toothpick method of inoculation was used in all subsequent experiments.

**Figure 2:**
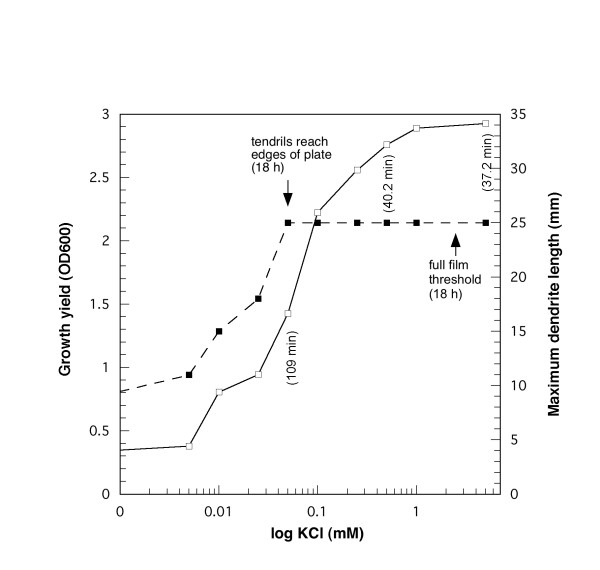
**Quantification of the K^+ ^requirement for *hag *growth on MSggN liquid and semisolid media**. Cultures of *hag *were grown in MSggN semisolid or liquid media with the indicated levels of KCl, each in triplicate (semisolid) or duplicate (liquid), at 37°C, and the results were averaged. For the semisolid medium, the effect of KCl concentration on the maximum tendril length as measured at 18 h () is shown; KCl thresholds for tendril growth and formation of films that covered the semisolid media (50 mm plate surface) are indicated. For liquid MSggN medium the growth yield () and doubling times (three KCl levels from the tendril threshold to above the full film threshold) are shown. The experiment was replicated in a separate experiment with essentially the same results.

To further investigate the K^+ ^requirements for the tendril and film growth phases, MSggN media were prepared with varying levels of KCl and the colony expansion on surfaces was compared to the growth rate in broth. As seen in Fig. 2, the KCl level in MSggN broth had dramatic consequences on *hag *growth such that growth yield was proportional to the amount of potassium ion in the medium; cell doubling times were also dependent on KCl concentration. No growth occurred if KCl was omitted. Similar KCl-dependent growth yields and cell doubling times were obtained in MSggN broth with the parent *B. subtilis *3610 strain (data not shown). The surface growth behavior of *hag *on MSggN agarose was similarly affected, in which the formation of tendrils that reached the edges of the plates was correlated with low KCl concentrations (up to 50 μM), whereas complete film coverage of the agarose surface required about 40 times that amount. *B. subtilis hag *sliding motility for the tendrils was substantial, typically 0.4–0.6 μm s^-1 ^depending on the level of K^+ ^ion and growth temperature. From these observations, we conclude that the different modes of surface colonization are correlated with K^+^-dependent growth rate. The stimulation of surface film growth by KCl could not be replaced by addition of other monovalent cation sources, including LiCl, RbCl, CsCl, or the relatively high level of Na^+ ^ion in the basal MSggN medium (data not shown).

### The role of high and low affinity potassium ion transporters in the hag sliding phenotypes

**Figure 3:**
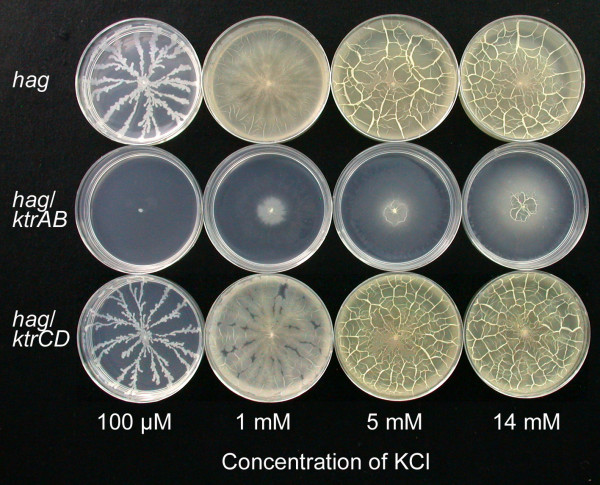
**The K^+^-dependence of *hag *growth on the defined medium requires the high affinity potassium transporter (KtrAB), but not the low affinity transporter (KtrCD)**. MSggN plates were prepared with the indicated levels of KCl, inoculated in triplicate with either the *hag *strain, or *hag ktrAB or hag ktrCD *triple mutants (Table 1), and photographed after 24 h growth at 37°C.

The finding that very low levels of K^+ ^ion are sufficient for the tendril growth seen in the *hag *strain suggested that this motility is dependent on the high affinity K^+ ^transporter (KtrAB), but not the low affinity K^+ ^transporter (KtrCD), present in *B. subtilis *[14]. To test this idea, we constructed the *hag ktrAB *triple mutant and as a control the *hag ktrCD *triple mutant. Each was grown on MSggN media with varying levels of KCl, with the results shown in Fig. 3. The results with the *hag ktrAB *mutant clearly indicate that disruption of the high affinity KtrAB transporter leads to inhibition of surface growth beyond the central point of inoculation of plates with 100 μM KCl, and weak central colony spreading at 1 to 14 mM KCl. In contrast, disruption of the low affinity KtrCD transporter resulted in a surface growth phenotype on MSggN plates that was indistinguishable from the parent *hag *strain. We have reported similar results with *ktrAB *and *ktrCD *mutations in *B. subtilis *3610, the wild-type parent of the *hag *strain examined here [15]. With respect to possible polarity effects, the *ktrAB *genes constitute a dicistronic operon ending in a putative rho-independent transcriptional terminator [16]. We therefore predict that the *ktrAB *insertion deletion construct is unlikely to have polar effects. While we have not tested complementation of the *ktrAB *alleles, these experiments are consistent with the importance of the KtrAB transporter for the growth phenotypes observed here, and further suggest that if tendril growth is disrupted by insufficient K^+ ^uptake surface films cannot form.

### The requirement of other media components for surface sliding motility

The dramatic effects of manipulation of K^+ ^ion levels on surface colonization in the defined medium led us to question if other components of the MSggN medium are essential for this type of sliding motility. Using the defined MSggN medium with KCl levels (5 mM) to satisfy the K^+^requirement, we investigated the effect of decreasing each macro- or micronutrient on the two phases of surface colony formation. These results are summarized in Table 2. First with respect to macronutrients, decreasing glycerol, Na-glutamate or Na-phosphate by factors of 10 each resulted in disruption of surface film growth, and tendril-like colonies formed. A further 10-fold decrease of these macronutrients led to tendril growth for lowered glycerol or Na-phosphate but poor growth for lowered Na glutamate; these results are indicative of thresholds for the nitrogen (glutamate), carbon (glycerol and glutamate) and phosphate sources of the MSggN medium. Substitution of MOPS buffer with either HEPES or PIPES buffer (all at pH 7.0) had no discernable effects on the surface growth patterns.

**Figure 4:**
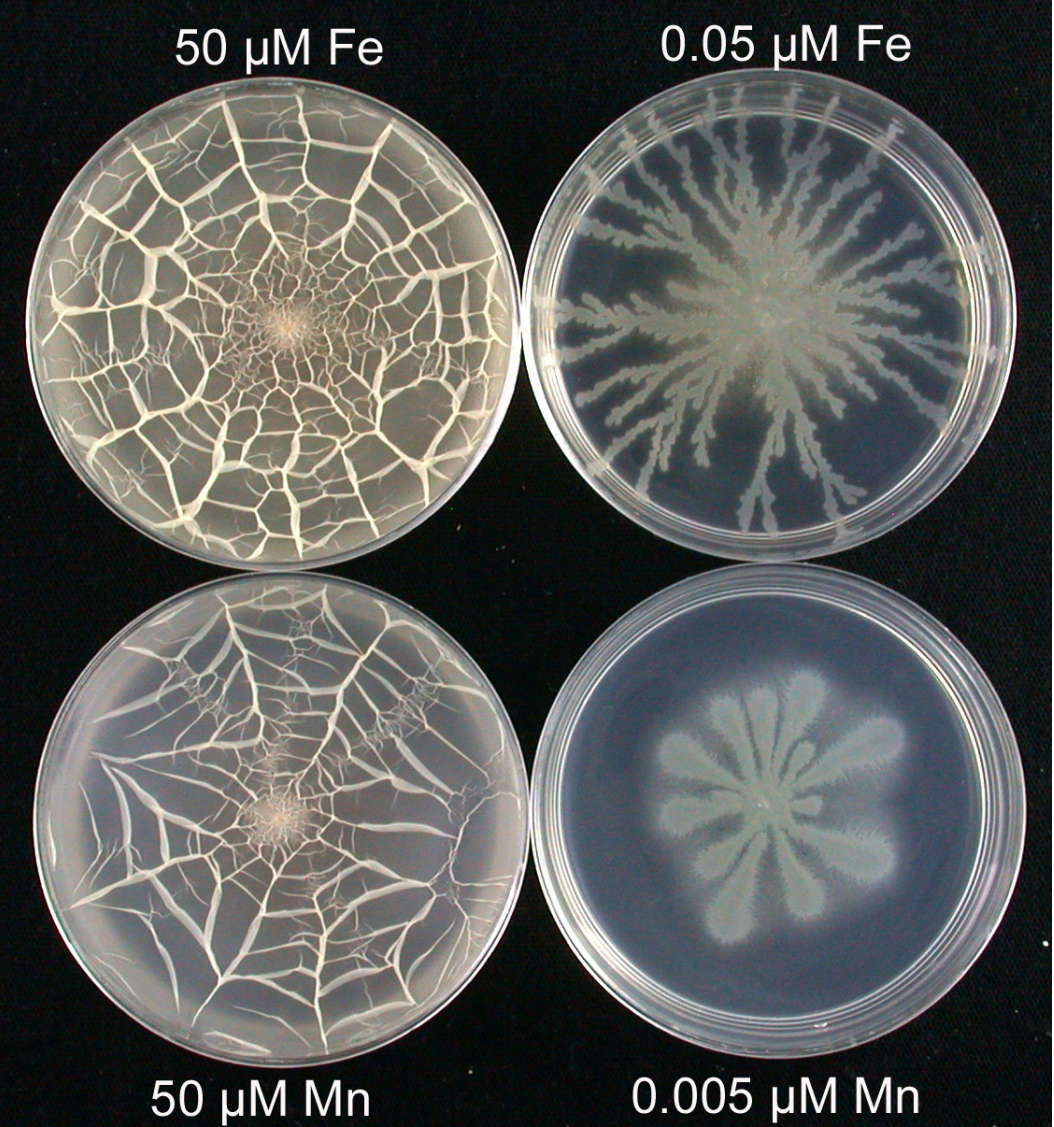
**Inhibition of surface film growth, but not tendril growth, occurs if either FeSO_4 _or MnSO_4 _is limiting**. In this experiment, replicated independently, MSggN plates (5 mM KCl) were prepared with the normal levels of FeSO_4 _or MnSO_4 _(50 μM), or with the decreased levels of FeSO_4 _or MnSO_4 _indicated. Plates were inoculated with the hag strain and photographed after 24 h growth at 37°C. The level of agarose in the MSggN plates with MnSO_4 _was raised to 0.4% w/v (normally 0.3% w/v) to accentuate the details of the tendrils.

When micronutrient levels were manipulated, it was seen that decreasing Mg^2+^, Fe^2+ ^and Mn^2+ ^eliminated the surface films but supported tendril growth, each with different thresholds: 20 μM MgCl_2_; 0.05 μM FeSO_4_; or 0.005 μM MnSO_4 _(Table 2). Even when diluted 10^-5^-fold, there was no effect of lowering CaCl_2 _or ZnSO_4 _(Table 2); surface films formed in each case. Some of the most striking visual results were obtained with lowered levels of Fe^2+ ^and Mn^2+ ^as shown in Fig. 4. These images show that at the threshold levels of these metals distinctly different tendril-like patterns of colony growth occur instead of complete surface film growth. Thus, as with K^+ ^ion limitation (Fig. 1), lowering other metals in the medium can produce generally similar surface growth phenotypes. In each case, tendril rather than film growth could be due to decreased growth rate produced by the limiting component of the medium. However, in the examples shown in Fig. 4 incubation of plates for up to 72 h led to persistence of tendrils without film formation (even when plates were incubated in a humid chamber to prevent drying).

### Examination of tendril morphology by confocal scanning laser microscopy (CSLM)

**Figure 5:**
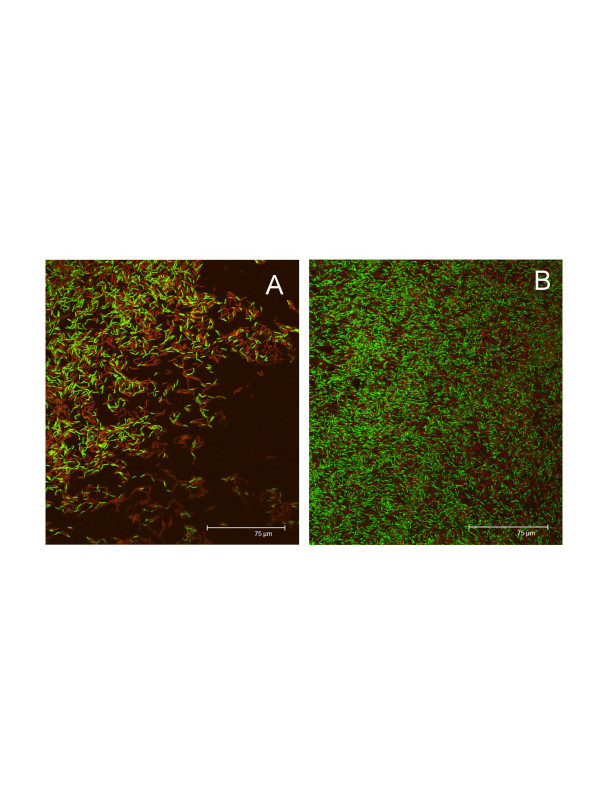
**Confocal scanning laser microscopy (CSLM) of *hag *tendrils**. In this experiment hag tendrils growing on MSggN plates (with 100 μM KCl) at 37°C were analyzed by CSLM using LIVE/DEAD BAcLight straining as described in the Methods. A) Tendril staining directly on a plate, where a 10 μl drop of stain (SYTO 9 and propidium iodide) was applied to a tendril and then covered with a cover slip (lightly pressed). B) Staining of a tendril that was removed from a plate by transfer to a cover slip that was then examined after contact with a 5 μl drop of stain on a glass slide.

To determine the organization of cells in during sliding of the *hag *mutant, we examined tendrils growing on MSggN agarose with low and high KCl, respectively, by CSLM using LIVE/DEAD BacLight straining methodology (Molecular Probes, Inc.; [17]). As shown in Fig. 5A, the staining of a tendril directly on the plate (under a cover slip) led to some dispersion of the tendril and an array of both live (green fluorescence) and dead (red fluorescence) cells. Nonetheless, the cells within the tendril did display cellular organization but were not as closely appressed or found in long chains as seen in *B. subtilis *pellicles [10]. The relatively high numbers of dead cells were likely due to the length of time the sample was examined in the CSLM instrument. In Fig. 5B, when tendril material was transferred to a coverslip and then quickly examined on a microscope slide, the tendril was much more intact and the majority of the cells imaged on the surface were live as evidenced by green fluorescence. Again, the cells did not appear to be forming closely-appressed, long chains. Attempts to image surface film colonies by these methods were complicated by the high density and thickness of the surface film. Clearly, more work is needed to understand the multicellular nature and cell viability of *hag *tendrils and films, but the CSLM images presented here support the idea that tendrils are indeed multicellular structures.

## Discussion

As is evident from many recent reviews (e.g. [1,18-20]), there is considerable interest in the mechanisms of bacterial motility on surfaces. Considering that *B. subtilis *is found in the soil, it is likely that cell populations of these bacteria utilize motility mechanisms to colonize surfaces such as plant roots [21]. Investigations of *B. subtilis *motility have primarily focused on flagellar-dependent mechanisms, including swimming and swarming behavior [22]. It is also notable that there are extensive investigations of pattern formation by *B. subtilis *on nutrient-limited agar media, a relatively slow and passive-type of motility supported by lubrication of the surface [[23,24], and references therein]. Here, we highlight the ability of *B. subtilis *to rapidly colonize surfaces using a flagellar-independent mechanism, sliding motility, and detail a defined medium to investigate this mode of surface migration.

We previously obtained evidence consistent with the phenomenon of sliding motility in wild type *B. subtilis *strains that produce the lipopeptide surfactin [9]. However, the conclusion that surface colonization was primarily due to flagella-independent motility rested on negative evidence, i.e. the failure to detect substantial numbers of flagellated cells at the moving boundary of spreading colonies. Here we have addressed this issue by studying surface spreading in a flagella-less mutant, in which the *hag *gene (encoding flagellin) was replaced by an antibiotic resistance cassette. As a result, any contribution of flagellar-dependent swarming motility can be eliminated. Notable features of the sliding motility of the *hag *mutant on MSggN agarose plates are i) that surface behavior of the *hag *mutant is comparable to the wild type, ii) colony spreading requires surfactin, iii) selective manipulation of nutrients alters growth and surface behavior, and iv) sliding migration is correlated with cellular growth rate.

Very few rates of sliding motility in bacteria have been published: 0.03–0.04 μm s^-1 ^for *M. smegmatis *[25] to rates as high as 6 μm s^-1 ^for *Serratia marcescens *[1]. *B. subtilis *sliding motility was substantial, typically 0.4–0.6 μm s^-1 ^depending on the level of K^+ ^ion and growth temperature. Because the rate of colony expansion depends on the rate of cell growth, the sliding behavior of different bacteria under different media conditions may not be directly comparable. Nonetheless, we note that the rates of *B. subtilis *sliding measured here fall within the published range for this type of surface behavior.

*B. subtilis *sliding proceeds in two distinct growth phases, tendril and film growth. Tendrils first radiate from the point of inoculation, and if nutrient levels are sufficiently high, film growth begins and fills in the voids between the tendril arms. The two phases of sliding are analogous to similar behaviors seen in *M. smegmatis *[25]. In *M. smegmatis*, growth on a semi-solid medium with low levels of nutrients can lead to the appearance of tendril-like extensions that spread outwards to the edges of the plate, whereas growth on semi-solid nutrient media is seen as a distinct spreading film. Unlike *M. smegmatis *however, *B. subtilis *exhibits both behaviors, tendril and film-like growth, sequentially on the same medium. The question remains, why, in the presence of high levels of nutrients, does *B. subtilis *produce tendrils first rather than initiating film migration immediately. We suggest that the two behaviors may have to do with the fact that surfactin, an extracellular lipopeptide required for both tendril and film formation, is regulated by quorum-sensing. Surfactin production is known to be activated by a pheromone called ComX [26]. As *B. subtilis *grows, it secretes the ComX peptide, extracellular ComX concentration increases proportionally with cell density, and at a critical cell density, ComX activates surfactin production. Therefore, tendril formation may be the manifestation of a population at subcritical cell-density that secretes surfactin at levels insufficient to permit film spreading. The tendril continues to grow, ComX accumulates, surfactin production increases, and film formation initiates. Consistent with the idea that tendrils form when surfactin is limiting, we have found that when small amounts of surfactin (50–200 μg) are added to the center of agarose plates prior to inoculation, *B. subtilis hag *cells bypass the tendril spreading phase and proceed directly to film formation (unpublished observations). The tendrils are multicellular assemblies of cells, as shown by confocal scanning laser microscopy (CLSM; Fig. 5), but the control(s) over their initiation at the point of inoculation are unknown.

The continued growth of the spreading surface colonies produced a web-like appearance (e.g. Fig. 1), with the formation of sheets of cells that often collapsed onto the agarose surface. It is notable that the web-like surface films seen here are similar in appearance to floating pellicles formed by undomesticated *B. subtilis *strains [10,13], and it is possible that the spreading of multicellular structures on semi-soft MSggN medium shares features with multicellular organization in pellicles. However, further microscopic examination is needed to investigate this point.

Lowering the levels of essential nutrients can block the transition from tendril growth to film growth or abolish sliding motility entirely. The mechanism of these effects was examined in more detail for the K^+ ^requirement, and we found that the K^+ ^threshold for tendril growth was very low, only 50 μM, while that for full surface film formation was in the range of 2–3 mM. Both of these thresholds can be explained by the uptake of K^+ ^ion by the KtrAB potassium transport system, which Holtmann et al. [14] have shown to be the major, higher affinity K^+ ^transporter in *B. subtilis*. The KtrAB system and a second lower affinity KtrCD system have K_m _values of approximately 1 mM K^+ ^and 10 mM K^+^, respectively [14]. The essential role of the KtrAB transporter in sliding motility was confirmed by the use of a *hag ktrAB *triple mutant. The model that emerges is that at K^+ ^concentrations far below the K_m _of the KtrAB transporter, *hag *cells can grow only in clusters in the tendril form, and the growth rate is greatly reduced. In contrast, at levels of K^+ ^ion above the K_m _of the KtrAB transporter full film growth can occur since these higher K^+ ^levels allow much more rapid metabolism and growth. This data supports the idea that potassium ion has an intracellular role in sliding motility and that growth rate and sliding motility are highly correlated. It is also likely that limitations of other essential metals, Mg^2+^, Fe^2+ ^and Mn^2+ ^(Table 2), inhibit growth rate, also resulting in the tendril phenotype. In addition, it is known that surfactin production in *B. subtilis *is greatly enhanced by the presence of iron sulfate [27], and perhaps at lowered levels of iron the surfactin formation needed to support robust film growth is insufficient.

## Conclusion

Overall, the results presented here describe a defined medium and flagella-less *hag *mutant to investigate sliding motility in *B. subtilis*. When presented with a balanced MSggN medium the *hag *mutant can rapidly colonize the entire surface – likely by conditioning the surface with surfactin and using the expansive force of growth to form robust films. Perhaps of greater interest is the finding that nutritional limitations, especially for metals such as K^+^, Fe^2+ ^or Mn^2+^, cause the surface growth pattern to revert to the tendril form, where clusters of cells exhibit organized sliding motility in distinctive patterns reminiscent of those described by many other investigators, but under very different and slow growth conditions (reviewed in [1]). We suggest that formation of the tendrils may represent a growth strategy for nutrient-deprived cells to colonize surfaces in natural environments, such as plant roots where large populations of *B. subtilis *and its relatives have been found (reviewed in [21]). It will be of interest to examine the controls on tendril formation and determine why the tendril phase persists and precedes the formation of surface films. These future experiments should also shed more light on the phenomenon of bacterial sliding motility.

## Methods

### Strains and media for the study of sliding motility

Table 1 summarizes the strains used in this work. Most of the experiments described here used *B. subtilis *DS64, a *hag *mutant (*hag*::MLS) constructed in wild type strain 3610, using gene replacement methods described elsewhere [5]. DS64 was routinely maintained on Luria-Bertani (LB) medium containing (per L) 10 g tryptone, 5 g yeast extract, and 10 g NaCl, solidified with 1.5% w/v agar (Difco) and supplemented with erythromycin (1 μg ml^-1^) and lincomycin (25 μg ml^-1^; referred to as MLS for macrolide-lincosamide-streptogamin B resistance). Verification of the lack of flagella in the *hag *mutant included examination by flagellar staining [28] and its inability to swim on LB agar plates (0.3% w/v agar; [5]). For surface sliding motility, the pellicle medium MSgg described by Branda et al. [10] was used with some modifications: 1) the 5 mM KH_2_PO_4 _component was replaced by 5 mM NaH_2_PO_4 _and K^+ ^ion was provided by addition of KCl as described below; 2) the metals solution, prepared as a separate 100× filter sterilized stock and stored at 4°C, was supplemented with 5 mM ascorbic acid to keep the iron component (5 mM FeSO_4_) from oxidizing and precipitating; and 3) thiamine, tryptophan and phenylalanine were deleted. This modified medium, MSggN, supported good planktonic growth (37°C) if 100 μM KCl was added, and weak pellicle formation in stationary cultures (30°C, 48–60 h) if 500 μM KCl was added; when supplemented with 5 mM KCl pellicle formation of strain 3610 and its *hag *mutant was robust with a thick wrinkled pellicle as in Branda et al. [13]. For sliding motility the MSggN medium, prepared with varying amounts of KCl, was solidified with 0.3% w/v agarose (Fisher Scientific; low EEO high gel strength grade). The 60 mm plates were allowed to air dry on a leveling table in a laminar flow hood overnight, and then routinely inoculated in the center with a sharpened toothpick from overnight cultures of DS 64 on LB (MLS) agar, or LB agar for its wild type parent strain 3610. In some experiments, plates were inoculated in the center from LB (MLS) broth cultures so that the inoculum (2.5 μl) contained 5 × 10^3^, 5 × 10^4^, or 5 × 10^5 ^viable cells. Following growth at 37°C for 16–24 h the MSggN plates were photographed. Except for pellicle growth (30°C), all other growth experiments were conducted at 37°C.

**Table 1:** *B. subtilis *strains used in this work

Strain	Genotype/phenotype	Reference/source
3610	Undomesticated wild type	NCIB 3610; [10]
JH642	*trpC2 pheA1*	J. Hoch
DS64	*hag*::MLS	[5]
GHB1	Δ(*ktrAB*::neo)	E. Bremer
GHB6	*ktrC*::spec	E. Bremer
GHB12	*ktrD*::tet	E. Bremer
M1	*srfAA*::cat	[9]
RFH1	*hag*::MLS *srfAA*::cat	This work
RFH3	*hag*::MLS Δ(*ktrAB*::neo)	This work
RFH7	*hag*:: MLS *ktrC*::spec *ktrD*::tet	This work

**Table 2:** Consequences of selective manipulation of MSggN media composition on surface colony formation of the *hag *null strain. This data shows how systematically lowering concentrations of macronutrients and micronutrients affects the growth pattern on MSggN agarose plates. In each case 5 mM KCl was present, except where KCl was varied, and plates were inoculated in triplicate to reveal the surface colony pattern (film or tendrils) after 24 h growth at 37°C.

Media component	1X conc.	0.1X conc.	0.01X conc.
*Macronutrients*			
54 mM Glycerol	Film	Tendril	Tendril
30 mM Na glutamate	Film	Tendril	Weak growth
5 mM NaH_2_PO_4_	Film	Tendril	Tendril
*Micronutrients*			
5 mM KCl	Film	Tendril	Tendril
2 mM MgCl_2_	Film	Film	Tendril^a^
700 μM CaCl_2_	Film	Film	Film^b^
50 μM MnSO_4_	Film	Film	Film^c^
50 μM FeSO_4_	Film	Film	Film^d^
1 μM ZnSO_4_	Film	Film	Film^b^

^a^Tendrils were also formed at the 10^-3 ^to 10^-5 ^dilutions.

^b^For these micronutrients, films were also formed at the 10^-3 ^to 10^-5 ^dilutions.

^c^Films were formed at the 10^-3 ^dilution and tendrils were formed at the 10^-4 ^and 10^-5 ^dilutions.

^d^Tendrils were formed at the 10^-3 ^to 10^-5 ^dilutions.

In some experiments, the levels of agarose, macro- or micronutrients were varied as described in the text (and Table 2). To examine the monobasic cation specificity of sliding motility, the KCl component was replaced with equimolar LiCl, RbCl or CsCl. For planktonic growth of the hag mutant, MSggN broth with varying levels of KCl were used, and cell density was determined from the optical density at 600 nm.

### Construction of srfAA, ktrAB and ktrCD mutants in the hag strain

Strains (*hag *genetic background) with disruptions of surfactin formation or high and low affinity K^+ ^uptake systems, KtrAB and KtrCD, were constructed, and are listed in Table 1. The surfactin mutant was derived from a *srfAA *mutant derived by gene disruption [9]. KtrAB and KtrCD, were constructed from mutants obtained from E. Bremer each in the laboratory strain JH642 [14]. Each allele was transferred to the *hag *strain (DS 64; *hag*::MLS) by SPP1-mediated phage transduction to obtain strains RFH1(*hag*::MLS *srfAA*::cat), RFH3 (*hag*::MLS Δ*ktrAB*::neo) and RFH7 (*hag*::MLS *ktrC*::spec *ktrD*::tet). Strains were typically maintained on LB agar supplemented with the appropriate antibiotics, 5 μg ml^-1 ^chloramphenicol, 10 μg ml^-1 ^neomycin, 10 μg ml^-1^tetracycline, or 100 μg ml^-1 ^spectinomycin. Strains were periodically checked for the MLS marker of the *hag *mutant, and stored for longer periods frozen in 7% (v/v) DMSO at -70°C.

### Confocal Scanning Laser Microscopy

To examine the colony morphology in both tendrils and surface films, confocal scanning laser microscopy (CSLM) was used. Surface colonies on plates or colonies transferred directly to a glass cover slip were stained with Live/Dead BacLight stains from Molecular Probes (Eugene, OR) following the general protocol provided by the manufacturer. In this case a kit 1:1 mixture of green-fluorescent SYTO 9 and red-fluorescent propidium iodide was diluted 5-fold with phosphate-buffered saline and used for staining. Stained cells were examined and images processed with a Leica TCS SP2 AOB5 microscope as described in detail in Lopez et al. [17]).

## Authors' contributions

RF carried out many of the aspects of the work and drafted the manuscript. DBK provided strains, guidance on further strain construction, and contributed to the final version of manuscript. TN conducted many of the experiments including the construction of the *hag ktrAB *and *hag ktrCD *triple mutants.
